# Experience of the use of Ketamine to manage opioid withdrawal in an addicted woman: a case report

**DOI:** 10.1186/s12888-016-1112-2

**Published:** 2016-11-10

**Authors:** Laurence Lalanne, Chloe Nicot, Jean-Philippe Lang, Gilles Bertschy, Eric Salvat

**Affiliations:** 1 Department of Psychiatry and Addictology, Fédération de Médecine Translationnelle de Strasbourg (FMTS), University Hospital of Strasbourg, Strasbourg, France; 2 INSERM 1114, Fédération de Médecine Translationnelle de Strasbourg (FTMS), University Hospital of Strasbourg, Strasbourg, France; 3 Centre d’Evaluation et de Traitement de la Douleur, University Hospital of Strasbourg, Strasbourg, France; 4 Institut des Neurosciences Cellulaires et Intégratives, Centre National de la Recherche Scientifique, Strasbourg, France

**Keywords:** Ketamine, Opioid addiction, Opioid withdrawal, Painkillers

## Abstract

**Background:**

Opioids are good painkillers, but many patients treated with opioids as painkillers developed a secondary addiction. These patients need to stop misusing opioids, but the mild-to-severe clinical symptoms associated with opioid withdrawal risk increasing their existing pain. In such cases, ketamine, which is used by anaesthetists and pain physicians to reduce opioid medication, may be an effective agent for managing opioid withdrawal.

**Case presentation:**

We describe the case of a woman who developed a severe secondary addiction to opioids in the context of lombo-sciatic pain. She presented a severe opioid addiction, and her physicians refused to prescribe such high doses of opioid treatment (oxycontin® extended-release 120 mg daily, oxycodone 60 mg daily, and acetaminophen/codeine 300 mg/25 mg 6 times per day). To assist her with her opioid withdrawal which risked increasing her existing pain, she received 1 mg/kg ketamine oral solution, and two days after ketamine initiation her opioid treatment was gradually reduced. The patient dramatically reduced the dosage of opioid painkillers and ketamine was withdrawn without any withdrawal symptoms.

**Conclusion:**

Ketamine displays many interesting qualities for dealing with all symptoms relating to opioid withdrawal. Accordingly, it could be used instead of many psychotropic treatments, which interact with each other, to help with opioid withdrawal. However, the literature describes addiction to ketamine. All in all, although potentially addictive, ketamine could be a good candidate for the pharmacological management of opioid withdrawal.

## Background

Opioid addiction affects 15.5 million people in the world, including 11.1 million heroin users (WHO) (United Nations Office on Drugs and Crime, 2007) [1]. Although in the USA, opioid prescriptions rose between 2002 and 2010 but have been decreasing since 2013, owing to physicians’ awareness of a rise in the opioid-related death rate and the increase in opioid medications [2]. Consequently, these patients need to end their misuse of opioids, but the mild to severe clinical symptoms associated with opioid withdrawal risk increasing the pain already present. Such symptoms are dysphoria, restlessness, rhinorrhea, lacrimation, myalgias, arthralgias, nausea, vomiting, abdominal cramps, diarrhea, yawning, increased flatulence and piloerection. Accordingly, in cases of severe somatic or psychiatric withdrawal symptoms, it might be preferable to manage opioid withdrawal when patients are hospitalized (Guidelines for the management of opioid withdrawal, WHO, 2009) [3]. There is a risk of complications such as distress, increased heart rate, high or low blood pressure. Anxiety, depression and, more rarely, psychotic episodes have been described when opioids are stopped suddenly [4–6]. Opiate withdrawal may be done gradually, using opiate substitution drugs such as methadone or buprenorphine, or abruptly. In the latter case, withdrawal symptoms are managed with adjunctive medication such as clonidine and benzodiazepine, and medication to treat the symptoms. Clonidine binds to a central alpha 2 adrenergic receptor that shares potassium channels with opioids and blunts withdrawal symptoms. In addition to clonidine, benzodiazepines and other GABAergic drugs reduce catecholamine release during severe withdrawal, and the benzodiazepines themselves have been shown to reduce withdrawal symptoms in animal models [7, 8]. Symptomatic medications such as loperamide, phosphoglucinol, and octreotide could be added for gastro-intestinal symptoms. Thus, managing opiate withdrawal requires multiple prescriptions, with a high risk of drug-drug interaction and incomplete patient relief, a particularly sensitive issue in the case of patients taking opioid drugs in the context of pain associated with somatic disease.

In such cases, it might be worth investigating whether ketamine, widely used by anaesthetists to limit opioid tolerance, reduce use of painkillers, and increase the time preceding the first request for painkillers during the postoperative period [9], may be efficient for managing opioid withdrawal. Ketamine has also shown it has a role to play as an anti-hyperalgesic and tolerance-protective drug. It is acknowledged that it is of interest for managing various pain conditions, such as pain connected with opioid tolerance, acute pain or chronic pain [10–12]. Pain physicians use ketamine at sub-anaesthetic doses to treat refractory chronic pain syndromes, especially cancer- and non-cancer-related pain, or pain with a neuropathic component [13–15]. Moreover, recent publications showed that use of alpha2-adrenoreceptor agonists such as clonidine and NMDA antagonists such as ketamine or dextromethorphan could minimize tolerance development during opioid treatment [16]. As well as the tolerance effect, it is well known that chronic opioid medication can potentially increase subjective pain when prescribed for a long period [17]. Chronic treatment with opioids may also be complicated by abnormal pain sensitivity such as opioid-induced hyperalgesia [18, 19]. In cancer pain patients who have lost an analgesic response to high doses of opioids, a synergistic effect between oral ketamine and opioids and a rightward shift of the opioid-response curve were observed [20].

Ketamine is classified as an NMDA receptor antagonist but has many other pharmacological actions on mu, delta, kappa opioid receptors and monoamine transporters, inhibiting serotonin, dopamine and norepinephrine reuptake. Low doses of ketamine reduce morphine use, nausea and vomiting after surgery [21, 22], an effect mediated by NMDA antagonism plus μ-opioid and sigma receptor activation. In the central nervous system, ketamine, like other NMDA receptor antagonists, triggers anesthetic, amnesic, dissociative, and hallucinogenic effects. Activation of κ-opioid receptors and possibly sigma and mACh receptors may also contribute to its psychotomimetic properties [21]. Dopamine and serotonin reuptake inhibition is likely to underlie an antidepressant effect which is clinically relevant in depressed patients [23], although an additional but weak action of μ-opioid receptor activation cannot be ruled out [21]. In peripheral systems, ketamine enhances catecholaminergic transmission, producing measurable changes in peripheral organ systems, including the cardiovascular, gastrointestinal, and respiratory systems [24], increasing sympathetic stimulation of the cardiovascular system, and reducing nausea and vomiting and respiratory complications like bronchoconstriction. However, ketamine side effects may induce aberrant percepts, including musical and auditory verbal hallucinations [25], and its repetitive administration may lead to the development of additive behavior [26, 27]. Accordingly, its use in medical care should be strictly supervised.

Some studies looked at the effect of ketamine for managing opioid withdrawal in the case of precipitated opiate withdrawal. One study, involving 58 patients who underwent rapid opiate antagonist induction under general anesthesia, showed that ketamine could help manage opioid withdrawal [28]. Prior to opiate antagonist induction, patients were given either a placebo (normal saline) or subanaesthetic ketamine infusion of 0.5 mg/kg/h. The ketamine group presented better control of withdrawal symptoms, lasting beyond the ketamine infusion itself. Significant differences were noted between the ketamine and control groups in the anaesthetic and early postanaesthetic phases, but no differences in effects were observed in terms of outcome after 4 months. In the same year, a Japanese team reported the case of a 2 year-old girl who received ketamine perfusion to manage symptoms caused by opioid withdrawal precipitated by naloxone [29]. To the best of our knowledge, there is no literature on ketamine use in opioid withdrawal not precipitated by naloxone. Here we report, according to the CARE (CAse REport) guidelines [30], the case of a 36 year-old woman hospitalized for opioid painkiller addiction who was treated with ketamine for the purpose of opioid reduction and withdrawal.

## Case presentation

In 2015, a 36 year-old woman was admitted to the rheumatology department of Strasbourg University Hospital (CHU) suffering from lumbar pain (or low back pain) with hyperalgesia and addiction to painkillers. A divorced mother of a child, the patient had been employed until 2008. Her medical history included chronic spinal pain over a one-year period, two cases of deep vein thrombophlebitis (2001), pulmonary embolism (2001), a transient ischaemic attack (2004) with negative blood test results, and an appendectomy (2008). Owing to the functional consequences of left wrist surgery following a work accident, she left her job and received disability benefits. In 2014, she received opioid treatment and 3 epidural infiltrations for lumbar and radicular pain. As an outpatient, she was treated with pregabalin combined with transcutaneous electrical nerve stimulation (TENS), but the hospital then lost track of her. Her psychiatric and addictological record contained reports of an episode of depression, a morphine overdose-induced coma, hospitalization for opioid withdrawal, and an addiction to tobacco. When admitted to the rheumatology department in 2015, her treatment consisted of diazepam 10 mg three times per day, hydroxyzine 100 mg twice daily, venlafaxine 150 mg in the morning, oxycontin® extended-release 60 mg twice daily, oxycodone 10 mg every 4 h, and acetaminophen/codeine 300 mg/25 mg 6 times per day. She abused oxycodone and codeine, and her physicians refused to prescribe opioid treatment in such high dosages. Her medical examination revealed lumbosacral pain with intermittent irradiation towards the posterior side of her thigh down to the top of her left knee, painful limitation of flexion, extension, right rotation of the spine, limited flexion in both directions. The patient also reported pain upon palpation of the left pelvic-trochanter region, and cellulalgia in the dorso-lumbar region. She was suffering from dysuria and constipation due to opioid treatment. Biological investigations, CT-scan and X-ray results were normal. A spinal MRI scan found lumbar degenerative disc disease (L4-L5 and L5-S1). Rheumatologists asked for a specialist opinion from a pain specialist, who, in view of the patient’s prior psychiatric history, decided to contact psychiatrists and addictologists for their opinion. The patient presented with a histrionic personality (eg. need to be the center of attention, dramatization within the therapeutic relationship, reactivity of mood, excessive sensitivity to criticism or disapproval, inappropriate seductive appearance, rapidly shifting emotional states that may appear superficial and rash decision-making according to DSM-5 [31]). She also presented with some borderline traits (eg. unstable interpersonal relationships alternating idealization/devaluation, identity disturbance with unstable sense of self, difficulty controlling anger, transient paranoid ideations). Given the patient’s psychiatric symptoms and the fact that she wanted to withdraw opioid treatment by herself and attempted to do so, the physicians proposed she be admitted to the hospital’s psychiatry department to be treated for opioid withdrawal with the administration of ketamine to manage lombo-sciatic pain. She was informed of the side effects of ketamine and of the need to follow the protocol correctly and to report any opioid withdrawal symptoms. Faced with the fact that no physician could prescribe such high dosages of opioid medication, the patient agreed to be transferred to the psychiatric department. There she received ketamine oral solution (5 mg/ml, magistral formulae) 1 mg/kg, and two days after ketamine initiation her opioid treatment was gradually reduced (10 % reduction in the initial dosage each two days). The patient reported no withdrawal symptoms (Clinical Opioid Withdrawal Symptoms score of 0/11 in the first and seconds week after the physician started reducing her opioid medication), pain or cravings while her opioid treatment was being reduced. She complained of no side effects of ketamine other than an unusual weakness. However, because of her borderline and histrionic traits, the reduction in opioid treatment took longer, and we had to extend the period of ketamine treatment. In fact, the patient’s need to be at the center of attention caused many problems with other patients and with nurses, who found her demands difficult to deal with. As she presented with emotional and paranoid reactions, the medical staff proposed a long weekend break, thus interrupting her care and delaying her treatment. Although we had to extend the hospitalization period and ketamine treatment, in the end ketamine withdrawal was achieved without withdrawal symptoms, and the patient was discharged from the hospital. She consulted her addictologist and psychiatrist. She now takes very small dosage of opioids for pain relief (codeine 50 mg three times per day). To control administration of the remaining opioid medication, it is administered at home by a nurse. Functional re-education was also organized.

## Discussion

In this case, we reported on withdrawal of opioid medication in a woman presenting opioid addictive disorders but also suffering from chronic lumbar pain that justified the use of painkillers. According to DSM-5 criteria [31], her opioid use disorder was severe, insofar as she could not stop using opioids and, although she tried to lower the dosage of opioid medication, she increased it each time she got out of the hospital. Moreover, she risked her life by overdosing on opioid medication. The decision to stop taking opioid painkillers was not a spontaneous request on the part of the patient but a decision she took when she realized that no physician would prescribe her such a high daily dose. In the literature, it has been shown that replacing opioid medication with a substitution treatment of buprenorphine and buprenorphine-naloxone is effective for chronic pain and offers patients a good degree of satisfaction [32, 33]. However, as buprenorphine is off-label and buprenorphine-naloxone was off-label in France at this time, these treatments should be proposed only after withdrawal has been shown to have failed. Accordingly, the patient had difficulty accepting the idea of withdrawing opioid medications but agreed to reduce the dosage and to combine it with treatment, such as ketamine, for her lumbar pain. Ketamine introduction was easy, and the treatment was well tolerated by the patient, apart from an unusual weakness. Under ketamine, there were no opioid withdrawal symptoms (no nausea, vomiting, abdominal cramps, diarrhea, yawning, piloerection, restlessness) during the 3 weeks of ketamine medication and reduced opioid dosage. This observation is consistent with those described in the literature and confirms that ketamine could help cope with symptoms of opioid withdrawal [34]. As the patient had a history of depressive disorders, and as she was treated with antidepressants (venlafaxine 150 mg daily), she was assessed daily for depressive disorders by a physician. She showed no increase in depressive symptoms. As suggested in our introduction, the action of the ketamine on the dopamine and serotonin neurotransmissions have an antidepressant effect [23], which has been very interesting in the present case for avoiding depressive disorders which might emerge in the event of opioid withdrawal. This antidepressant effect was also interesting for avoiding a relapse in the patient’s opiate addiction, which most of the time is due to depressive behaviors induced by opioid decrease or withdrawal.

In our present case report, we showed that it is possible to use ketamine to reduce and almost withdraw opioid medications in the case of painful situations. We did not precipitate opioid withdrawal with naloxone before ketamine initiation, but, for the first time, ketamine was initiated just before opioid reduction. In this way, the combination of ketamine and opioid treatments reduced lumbosacral pain, and the use of ketamine counteracted withdrawal signs due to opioid reduction. Furthermore, we showed that ketamine could help patients lower their consumption of opioid medication with good tolerance levels, even if they present addictive disorders. It could be another way of dealing with opioid withdrawal symptoms in patients suffering from opioid addictive behaviors which are often an obstacle for them. Moreover, opioid withdrawal symptoms are currently managed with polytherapy based on benzodiazepine, hydroxyzine, antipsychotics, clonidine and many symptomatic treatments for nausea, vomiting, diarrhea, etc. There is uncertainty surrounding these associations, which are sometimes also insufficient for dealing with dysphoric symptoms, which are a main cause of relapse [5]. That is why ketamine’s long-lasting antidepressant effects could be very helpful during opioid withdrawal [23]. However, our case report is very complex given that the patient was prescribed many psychotropic and antidepressant medications, and it is difficult to conclude whether ketamine alone could counter all physical and psychological withdrawal symptoms in this case. Moreover, the patient’s histrionic personality might affect the successful use of ketamine due to her suggestibility. Since she received special treatment in a psychiatric unit, she was the center of attention during her spell in hospital. This attention probably made her feel she was a very special patient. This situation led to a brief interruption in her hospitalization and prolonged the ketamine treatment and care, such that she did not have to confront her own issues. To assess whether it is worth using ketamine to withdraw opioid medication, it may be necessary to study this effect in a clinical trial.

## Conclusion

All in all, ketamine displays many interesting qualities for dealing with all physical and psychological symptoms relating to opioid withdrawal. Accordingly, we propose that ketamine could be used instead of many psychotropic treatments for helping with opioid withdrawal, and, in this way, might be safer for patients suffering from addictive behaviors. However, the literature also describes addiction to ketamine [26, 27], which is why its use in patients suffering from addictive behaviors must be subject to stronger and stricter criteria. With this risk in mind, we propose testing ketamine medication for dealing with opioid withdrawal in the case of opioid addictive disorders in a “proof of concept” open study.
